# Elevation of cancer antigen 15-3 owing to oncocytic renal neoplasm in a patient without evidence of breast cancer recurrence on follow-up: a case report

**DOI:** 10.1186/s13256-023-04088-5

**Published:** 2023-12-07

**Authors:** Samuel G. Cockey, Hongzhi Xu, Mohammed Al-Jumayli

**Affiliations:** 1 University of South Florida Morsani College of Medicine, Tampa, FL USA; 2 Pathology, Moffitt Cancer Center, Tampa, FL USA; 3 Oncology Program, Moffitt Cancer Center, Tampa, FL USA

**Keywords:** Cancer antigen 15-3, Oncocytic renal neoplasm, Elevated tumor marker, Breast cancer, Case report

## Abstract

**Background:**

Cancer antigen 15-3 is a protein that clinicians commonly measure to monitor outcomes and response to treatment in patients with breast cancer. However, cancer antigen 15-3 can also be elevated in other, benign and malignant conditions.

**Case presentation:**

A 73-year-old White woman with history of breast cancer presented to her primary care physician with right hip pain, and laboratory testing revealed elevated cancer antigen 15-3. Further workup with radiographic imaging revealed a large mass in her right kidney. The renal mass was subsequently removed, and the cancer antigen 15-3 level returned to normal.

**Conclusions:**

Elevation of cancer antigen 15-3 owing to causes other than breast cancer recurrence can be a potential diagnostic pitfall during a patient’s follow-up. It is important for clinicians to be aware of the limitations of cancer markers and to utilize a combination of diagnostic tests for patient evaluation.

## Background

Tumor marker 15-3, also known as cancer antigen 15-3, is a protein that is often elevated in the blood of individuals with breast cancer. It is used as a marker to monitor the effectiveness of treatment and to detect recurrence of the disease. However, it has been known to be elevated in benign conditions and can result in false positives.

## Case presentation

A 73-year-old White woman with a remote history of stage IA triple-positive breast cancer in her right breast was diagnosed 5 years ago. She underwent lumpectomy and sentinel lymph node biopsy. Her diagnostic pathology was estrogen receptor (ER) 95%, progesterone receptor (PR) 92%, human epidermal growth factor receptor 2 (HER2) positive [immunohistochemistry (IHC) 3+] pT1cN0. She received adjuvant trastuzumab and paclitaxel for 3 months and adjuvant radiation, followed by adjuvant trastuzumab for 9 months.

Four years later, the patient’s primary care physician ordered a magnetic resonance imaging (MRI) examination of the right hip due to right hip pain. It was unremarkable except for osteoarthritis. He also ordered CA 15-3 and noted an elevated level of 293.9 U/mL (normal range 0–30 U/mL).

**Fig. 1:**
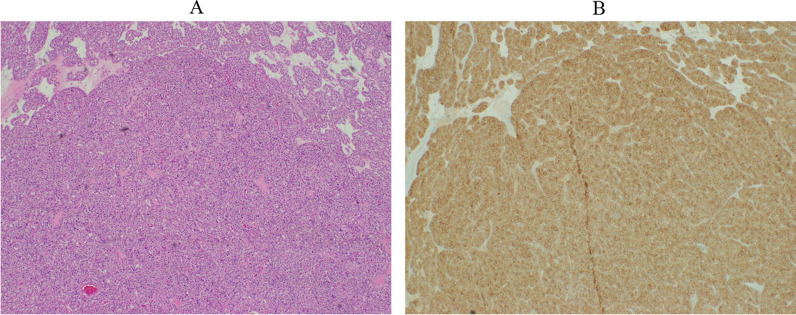
Oncocytic renal neoplasm pathology. **A** Hematoxylin and eosin stain. **B** Immunohistochemistry showing diffusely positive cancer antigen 15-3 stain on tumor cells

**Fig. 2:**
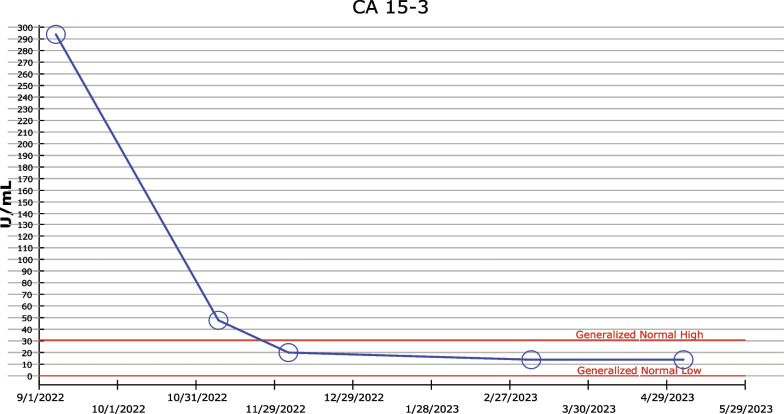
Graph of the serum cancer antigen 15-3 levels over time. Each circle represents a measurement. The upper and lower horizontal, dashed red lines represent the generalized normal high and low values of cancer antigen 15-3, respectively

The patient was referred to a medical oncologist for her abnormal elevated tumor markers. Other than mild joint pain, her medical history was unremarkable. The physical examination was pertinent only for an old healing scar from the right lumpectomy. Mammography with ultrasound and subsequent MRI of the breast revealed only postsurgical changes. Computed tomography (CT) of the abdomen/pelvis and whole-body positron emission tomography (PET) scan revealed a hypermetabolic 11.2 × 9.9 × 9.2 cm^3^ large enhancing mass within the mid and lower poles of the right kidney, highly suggestive of renal cell carcinoma. Based on this finding, she was referred to a urologic surgeon and subsequently underwent right radical nephrectomy. The surgical pathology revealed complete excision with clear margins. On postoperative histologic examination, the tumor was composed of a uniform population of plump cells with solid and nested growth patterns. The cells had eosinophilic cytoplasm and nuclei with variations in size and shape. Of note, some nucleoli were prominent. These features are characteristic of an oncocytic renal neoplasm with unknown malignant potential. IHC with an antibody to CA 15-3 resulted in diffuse labeling of the tumor cells (Fig. 1). Three months after surgery, the CA 15-3 concentration was within the normal range at 19.8 U/mL (Fig. 2).

## Discussion and conclusions

CA 15-3 is a protein that is found on the surface of many normal epithelial cells and that has been linked to changes in metabolism in cancer cells [1]. It is composed of two subunits that remain linked and has a heavily glycosylated outer domain. CA 15-3 may be useful in predicting the outcome and response to treatment in breast cancer [2, 3], but it is not typically used to monitor asymptomatic breast cancer follow-up [4].

Despite the usefulness of CA 15-3 for monitoring breast cancer recurrence, physicians must realize that other conditions can cause high CA 15-3 levels in patients without active breast cancer. Table 1 summarizes case reports of patients with or without history of breast cancer who showed elevated CA 15-3 in the absence of active breast cancer. In patients with history of breast cancer that was resolved, causes for the elevated CA 15-3 included pernicious anemia [5], normal pregnancy [6], and hypothyroidism [7]. Like the patient in the present case report, the patients in the three cited case reports showed normal CA 15-3 levels after receiving treatment for the cause of their CA 15-3 elevation.

**Table 1:** Previous reports of elevated cancer antigen 15-3 in the absence of active breast cancer

Authors and year	CA 15-3 level (U/mL)	Breast cancer history	Disease context	Ref.
Adachi *et al.* 2015	80	Yes	Pernicious anemia	[5]
Buonomo *et al.* 2019	92.6	Yes	Normal pregnancy	[6]
Aguiar-Bujanda *et al.* 2004	65.50	Yes	Hypothyroidism	[7]
Ghnassia *et al.* 2001	106	No	Renal oncocytoma	[8]
Bevan *et al.* 2016	448	No	Systemic lupus erythematosus	[9]
Tsiodras *et al.* 2018	73.9	No	Ovarian sarcoidosis	[10]
Altube Urrengoetxea *et al.* 2007	560	No	Idiopathic pulmonary fibrosis (male patient)	[11]

This table includes case reports of patients with or without history of breast cancer who showed elevated CA 15-3 in the absence of active breast cancer

CA 15-3 levels can also be elevated in patients without history of breast cancer and in the absence of active breast cancer. In our patient, an oncocytic renal neoplasm caused the elevated CA 15-3 levels in a patient with history of breast cancer. A previous case report noted elevated CA 15-3 level owing to a benign renal oncocytoma but in the absence of history of breast cancer [8]. After removing the affected kidney, the CA 15-3 levels returned to normal. Another case report showed elevation of four tumor markers, including CA 15-3, owing to systemic lupus erythematosus [9]. After treatment, this patient’s CA 15-3 level reduced but remained above the normal range. Other case reports showed elevated CA 15-3 levels due to ovarian sarcoidosis [10] or idiopathic pulmonary fibrosis [11] but did not report follow-up CA-15-3 values.

CA 15-3 is expressed in healthy kidneys [12], which may contribute to the CA 15-3 expression in the kidney tumor found in our patient. More specifically, researchers observed CA 15-3 expression in the distal tubules and collecting ducts of the nephrons of healthy kidneys but not in the proximal tubules [12]. Renal cell carcinomas also commonly express CA 15-3 [12]. As mentioned above, a renal oncocytoma in a patient without history of breast cancer also expressed CA 15-3 [8].

The most recent American Society of Clinical Oncology (ASCO) guidelines for the use of biomarkers in breast cancer therapy [13] and a recent retrospective analysis of CA 15-3 use for monitoring breast cancer relapse [14] do not suggest using CA 15-3 alone for therapy decisions or monitoring relapse, respectively. In 2015, the ASCO recommended using CA 15-3 only as an adjuvant assessment with respect to making decisions for therapy for metastatic breast cancer [13]. Similarly, the retrospective analysis concluded that the primary methods for monitoring breast cancer relapse should include the patient’s history, physical examination, and imaging [14]. The study found that patients with elevated CA 15-3 levels and symptoms of nausea, myalgia, or axial bone pain had a higher risk of breast cancer relapse [14]. Thus, this study recommends using CA 15-3 to supplement other parts of the patient’s case when monitoring for relapse [14]. However, it does not recommend using CA 15-3 levels alone because the levels can be elevated in the absence of active breast cancer, especially in asymptomatic patients or patients with a body mass index greater than or equal to 25 [14].

This case report includes strengths and limitations. To our knowledge, we are the first to report a patient with elevated CA 15-3 levels due to an oncocytic renal neoplasm in a patient without evidence of breast cancer recurrence on follow-up. While we only report one patient’s results, we believe that this case could be generalizable to other patients and can remind physicians that benign conditions can also cause elevated CA 15-3.

In conclusion, the patient’s elevated tumor marker CA 15-3 led to the detection of a renal tumor. This case illustrates that elevation of CA 15-3 can be due to causes other than breast cancer recurrence and can be a potential diagnostic pitfall during a patient’s follow-up. It is important for clinicians to be aware of the limitations of cancer markers and to utilize a combination of diagnostic tests for patient evaluation.

## Data Availability

The datasets used in the current study are available from the corresponding author on reasonable request.

## Abbreviations

ASCO: American Society of Clinical Oncology
CA: Cancer antigen
CT: Computed tomography
ER: Estrogen receptor
HER2: Human epidermal growth factor receptor 2
IHC: Immunohistochemistry
MRI: Magnetic resonance imaging
PET: Positron emission tomography
PR: Progesterone receptor
